# The relative contributions of the p53 and pRb pathways in oncogene-induced melanocyte senescence

**DOI:** 10.18632/aging.100051

**Published:** 2009-05-16

**Authors:** Sebastian Haferkamp, Sieu L Tran, Therese M Becker, Lyndee L Scurr, Richard F Kefford, Helen Rizos

**Affiliations:** Westmead Institute for Cancer Research and Melanoma Institute of Australia, University of Sydney at Westmead; Millennium Institute, Westmead Hospital, Westmead NSW 2145, Australia

**Keywords:** Oncogene-induced senescence, melanocytes, p53, pRb, p16, p21

## Abstract

Oncogene-induced senescence acts as a barrier against tumour formation and has been implicated as the mechanism preventing the transformation of benign melanocytic lesions that frequently harbour oncogenic B-RAF or N-RAS mutations. In the present study we systematically assessed the relative importance of the tumour suppressor proteins p53, p21^Waf1^, pRb and p16^INK4a^ in mediating oncogene-induced senescence in human melanocytes. We now show that oncogenic N-RAS induced senescence in melanocytes is associated with DNA damage, a potent DNA damage response and the activation of both the p16^INK4a^/pRb and p53/p21^Waf1^ tumour suppressor pathways. Surprisingly neither the pharmacological inhibition of the DNA damage response pathway nor silencing of p53 expression had any detectable impact on oncogene-induced senescence in human melanocytes. Our data indicate that the pRb pathway is the dominant effector of senescence in these cells, as its specific inactivation delays the onset of senescence and weakens oncogene-induced proliferative arrest. Furthermore, we show that although both p16^INK4a^ and p21^Waf1^ are upregulated in response to N-RAS^Q61K^, the activities of these CDK inhibitors are clearly distinct and only the loss of p16^INK4a^ weakens senescence. We propose that the ability of p16^INK4a^ to inhibit the cyclin D-dependent kinases and DNA replication, functions not shared by p21^Waf1^, contribute to its role in senescence. Thus, in melanocytes with oncogenic signalling only p16^INK4a ^can fully engage the pRb pathway to alter chromatin structure and silence the genes that are required for proliferation.

## Introduction

Fewer than 5% of patients with distant visceral metastases from cutaneous melanoma survive 12 months and there are no effective drug treatments [1]. The early molecular steps in formation of melanoma are therefore the subjects of intense scrutiny. Cutaneous melanoma arises from benign melanocytic lesions (benign naevi) or de novo from melanocytes of the skin [2]. Mutations activating the N-RAS or B-RAF kinase components of the mitogen-activated protein kinase (MAPK) pathway are found in approximately 15% and 60% of human melanomas, respectively [3-5].  Greater than 89% of B-RAF mutations in melanoma alter a single amino acid (V600E and V600K), whereas highly recurrent mutations affecting Gly-12, Ala-18 and Gln-61 account for approximately 12%, 5% and 70% of melanoma-associated N-RAS mutations, respectively [6]. The B-RAF^V600E^and N-RAS^Q61K ^mutations are also found in up to 80% and 55% of benign naevi, respectively [7,8] and benign naevi display several markers of senescence, including positive senescence-associated β-galactosidase (SA-β-Gal) activity and p16^INK4a^ expression [9,10]. Although the presence of senescent cells in human benign naevi remains controversial [11], accumulating evidence suggests that senescence occurs *in vivo* and acts as an effective barrier to tumour formation (Reviewed in [12]). Defining the relationship between oncogene activation, melanocyte senescence and escape from senescence remains an essential step in understanding melanomagenesis. For this reason we have sought to dissect the regulation of senescence in melanocytes.

The senescence program is established and maintained by the p53 and p16^INK4a^/retinoblastoma (pRb) tumour suppressor pathways. p53 engages a formidable proliferative arrest primarily in response to DNA-damage checkpoint signals triggered by telomere dysfunction and activated oncogenes [13-16]. For instance, the stable knockdown of p53-regulators (including ataxia telangiectasia mutated (ATM) and checkpoint-2 (CHK2) kinases) or p53 itself overcame RAS-induced sensecence in BJ human foreskin fibroblasts [15] (Table 1). Similarly, inactivation of the upstream p53 activator, ARF (p19ARF in mouse and p14ARF in human), overcame oncogene-induced senescence in mouse embryo fibroblasts (MEFs) [17,18], and loss of p21^Waf1^, a CDK inhibitor, activator of pRb and critical down-stream target of p53 transactivation, caused cells to bypass telomere-dependent replicative and oncogene-induced senescence in normal human fibroblasts and MEFs, respectively (Table 1) [19-21].

Although inactivation of the p53 pathway can reverse the senescence in some cells, there is an emerging consensus that it fails to do so in cells with an activated p16^INK4a^/pRb pathway [14,22,23]. Active, hypo-phosphorylated pRb interacts with E2F transcription factors and facilitates chromosome condensation at E2F target promoters. The reorganization of chromatin leads to the formation of senescence associated hetero-chromatin foci (SAHF) and the stable repression of E2F target genes that are involved in the irreversible cell cycle arrest associated with senescence [24]. Each SAHF contains portions of a single condensed chromosome, which is enriched for common markers of heterochromatin, including HP1γ, histone H3 methylated at lysine 9 (H3K9Me) and the non-histone chromatin protein, HMGA2 (reviewed in [25])

p16^INK4a^ is a positive regulator of pRb, via cyclin dependent kinase inhibition, and is crucial in generating SAHF [24]. Not surprisingly, p16^INK4a^ also acts as a tumour suppressor and is frequently inactivated in established human tumours. Inherited inactivating mutations in p16^INK4a^ are associated with melanoma susceptibility in melanoma-dense kindreds [26]. In fact, p16^INK4a^-deficient human melanocytes, derived from melanoma affected individuals, show an extended lifespan and are immortalized by ectopic expression of telomerase reverse transcriptase, whereas normal melanocytes display neither of these features [27,28]. Furthermore, replicative and oncogene-induced senescence are accompanied by accumulation of p16^INK4a^ in primary human cells [29-31] and ectopically expressed p16^INK4a^ initiates a senescence program characterized by cell cycle arrest, senescence-associated changes in cell morphology, increased SA-β-Gal activity and the appearance of SAHF [32,33].

The senescent states induced by the p53 and pRb pathways may be distinct and whether cells engage one or the other pathway appears to reflect the type of stress signal, the tissue and species of origin.  The relative contribution of the p53 and p16^INK4a^/pRb pathways in melanocyte senescence remains unclear, and recent data suggest the possibility of p53- and pRb-independent senescence pathways in these cells. For instance, N-RAS induced melanocyte senescence was associated with the activation of the p16^INK4a^/pRb and p53 pathways, but did not require expression of p16^INK4a^ or p14ARF [34].  Similarly, neither p53 nor p16^INK4a^ were required for H-RAS induced senescence in human melanocytes. Instead, H-RAS-driven senescence was mediated by the endoplasmic reticulum-associated unfolded protein response [35]. In another report, senescence induced by B-RAF^V600E^ or N-RAS^Q61R^ did not depend on p16^INK4a^ or p53 but could be partially overcome by expression of the oncogenic transcription factor c-MYC [36]. In contrast, p53 was found to be one of 17 genes (also included IGFBP7) required for BRAF^V600E^-mediated senescence of human melanocytes and p53 was also required for the induction of p16^INK4a^ following B-RAF^V600E^ expression [37] (Table 1).

In this study we systematically assessed the relative importanceof the tumour suppressor proteins p53, p21^Waf1^, pRb and p16^INK4a^ in mediating oncogene-induced senescence in human melanocytes. We confirm that N-RAS^Q61K^ induced senescence in melanocytes is associated with DNA damage, a potent DNA damage response and the activation of both the p16^INK4a^/Rb and p53/p21^Waf1^ tumour suppressor pathways. In melanocytes, the pRb pathway was the dominant effector of senescence, as its specific inactivation delayed the onset of senescence and weakened oncogene-induced proliferative arrest, as shown by the reduced formation of SAHF. Although p53-deficient melanocytes underwent a senescence response that was indistinguishable from that seen in wild-type melanocytes, the p53 pathway did contribute to the senescence program. In particular, the p53 pathway initiated a delayed arrest in pRb-deficient melanocytes, whereas melanocytes lacking both p53 and pRb continued to proliferate in response to oncogenic N-RAS. We also showthat, although p21^Waf1^ and p16^INK4a^[34] are not required for N-RAS induced senescence, both can activate pRb and promote senescence but only p16^INK4a^ triggers chromatin reorganization and the formation of SAHF. These data help to explain the observation that whereas p16^INK4a^ mutations are common in human cancer, p21^Waf1^ mutations occur rarely [38].

**Table 1.** Requirements of oncogene-induced senescence in human and mouse cells. *Not required*, gene expression is dispensable for oncogene-induced cell cycle arrest and senescence. *Required*, loss of gene expression overcame oncogene-induced cell cycle arrest. ^1^IMR90 cells senesce with longer telomeres and have higher basal levels of p16^INK4a^ than BJ cells [64, 73]. ^2^Fibroblasts from melanoma prone individuals with germline mutations inactivating p16^INK4a^. ^3^Loss of gene expression delayed or reduced oncogene-induced cell cycle arrest or SA-β-Gal activity. ^4^Loss of gene expression reduced oncogene-induced formation of SAHF. ^5^Overexpression of gene partially suppresses oncogene-induced SA-β-Gal activity. ^6^IL-6 expression is induced by oncogenic B-RAF in human melanocytes.

	**Human Cells**							**Mouse Cells**	
	*IMR90 Lung Fibroblasts^1^*		*BJ ForeskinFibroblasts^1^*		*Fibroblasts from melanoma-prone individuals^2^*	*Melanocytes*		*MEFs*	
**p53-DNA damage response**									
1. ATM	Required[16]/Not required[61]		Required [15, 16]		Not studied	Not studied		Not studied	
2. Chk2	Not studied		Required [15]		Not studied	Not studied		Not studied	
3. p53	Partial^3^[62]/ Not required [24, 29, 61, 63]		Required [15, 64]/ Partial^3 ^[62]		Not studied	Required [37]/ Not required [35, 36]		Required [29]	
4. ARF	Not required [65]		Not required [64]		Not required [66]	Not required [34]		Required [18, 67]	
5. p21^Waf1^	Not required [63]		Not studied		Not studied	Not required (this work)		Not required [21]	
**pRb pathway**									
1. pRb	Partial^3,4 ^[24, 62]/ Not Required [61]		Partial^4^[62]/ Not required[64]		Not studied	Partial^3,4 ^(this work)		Not required [45, 68]	
2. p107	Not studied		Not studied		Not studied	Not studied		Not required [68]	
3. pRb and p107	Not studied		Not studied		Not studied	Not studied		Required [68]	
4. p107 and p130	Not studied		Not studied		Not studied	Not studied		Not required [45]	
5. pRb, p107 and p130	Not studied		Not studied		Not studied	Not studied		Required [45, 68]	
6. p16^INK4a^	Partial^4^[24]/ Not required [16]		Partial^3 ^[15] / Not required [64]		Required[39, 66, 69, 70]/ Not required [71]	Partial^4^[34]/ Not required [35, 36]		Required [29]/ Not required [18]	
**p53 and pRb**	Required [61, 62]		Required [62]		Not studied	Required (this work)		Required [29]	
**p53- and pRb-independent**									
1. ER-stress response	Not studied		Not studied		Not studied	Required [35]		Not studied	
2. IL-6	Required [72]		Not studied		Not studied	Not studied^6 ^[72]		Not studied	
3. IGFBP7	Not studied		Not studied		Not studied	Required [37]		Not studied	
4. C-MYC	Not studied		Not studied		Not studied	Partial^5 ^[36]		Not studied	

## Results

**Figure 1.**
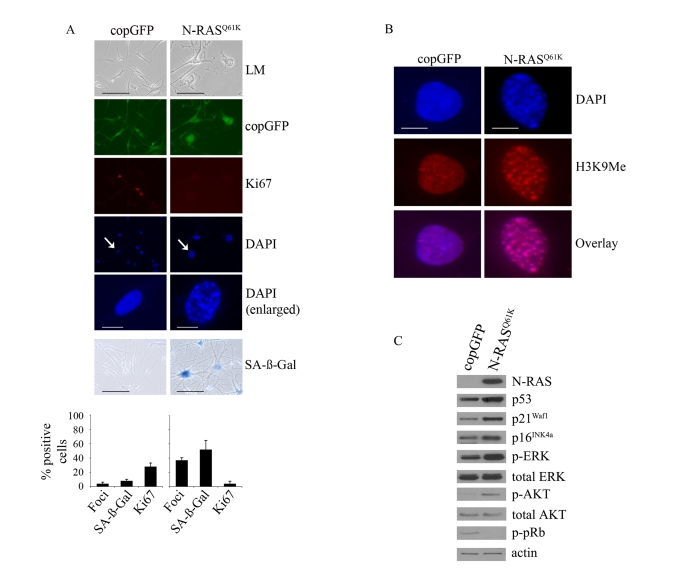
**Oncogenic N-RAS^Q61K^ induces proliferative arrest and senescence of human melanocytes.** (**A**) Human melanocytes were transduced with lentiviruses expressing N-RAS^Q61K^ or copGFP control. The efficiency of transduction was controlled with the co-expression of copGFP and was consistently above 90%. Cell proliferation (Ki67), chromatin condensation (DAPI), and the appearance of increased SA-β-Gal activity were analyzed and quantitated 15 days after infection. Percentage of cells positive for the indicated marker is shown in histograms, which correspond to the mean ± s.d. of at least two independent transduction experiments from a total of at least 300 cells. Cells enlarged to show DAPI-stained chromatin foci are indicated with arrows (bar =10 μm). LM, light microscopy (bar=100μm). (**B**) Human epidermal melanocytes infected with lentiviruses expressing N-RAS^Q61K^ or copGFP were stained with DAPI and antibodies to H3K9Me, 15 days post transduction (bar =10 μm). (**C**) Expression of the indicated proteins was determined by western blot analysis 15 days after infection of human epidermal melanocytes with lentiviruses expressing N-RAS^Q61K^ or copGFP control.

The response of primary human melanocytes to the oncogenic, melanoma-associated N-RAS^Q61K^ mutant was evaluated by stably transducing N-RAS^Q61K^ into human epidermal melanocytes. Accumulation of N-RAS^Q61K^ was detected three days post-transduction and the impact of N-RAS on melanocyte proliferation was monitored over 15 days. As expected, 15 days post-transduction the majority of N-RAS^Q61K^ transduced melanocytes displayed  several markers of oncogene-driven senescence, namely cell flattening, increase in cellular size, significantly reduced Ki67 expression, increased SA-β-Gal activity and the formation of SAHF (Figure 1A). As expected these foci were enriched for histone H3 methylated at lysine 9 (H3K9Me), a common marker of heterochromatin [24] (Figure 1B). In contrast, melanocytes accumulating the co-expressed Copepod GFP (copGFP) did not arrest, showed no evidence of chromatin condensation nor increased SA-β-Gal activity (Figure 1A).

**Figure 2.**
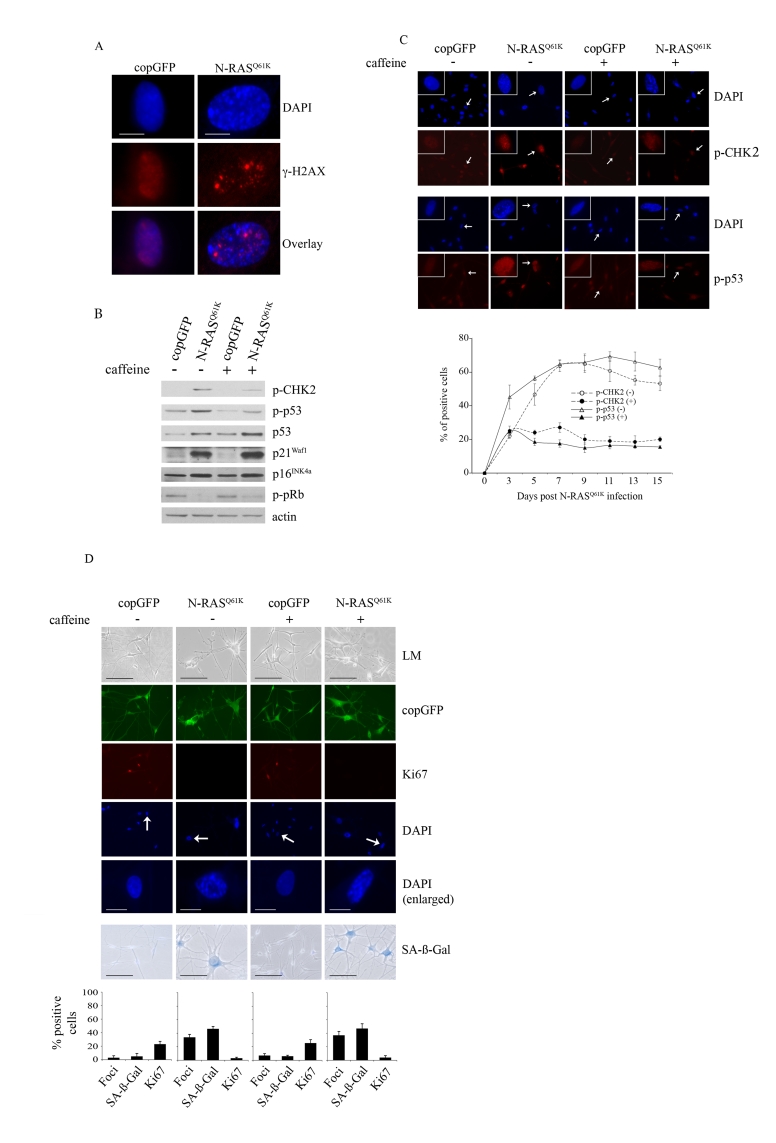
Oncogenic N-RAS ^Q61K^ induces DNA damage response in human melanocytes. (**A**) Human epidermal melanocytes infected with lentiviruses expressing N-RAS^Q61K^ or copGFP were stained with DAPI and antibodies to the DNA damage marker γ-H2AX, 15 days post transduction (bar =10 μm). (**B**) Human melanocytes were transduced with lentiviruses expressing N-RAS^Q61K^ or copGFP and cultured for 15 days in the presence (+) or absence (-) of 4mM caffeine. Expression of the indicated proteins was determined by western blot analysis 15 days after infection. (**C**) Melanocytes transduced with lentivirus expressing N-RAS^Q61K^ or copGFP and cultured for 15 days in the presence (+) or absence (-) of 4mM caffeine were stained with DAPI and antibodies against the phosphorylated forms of p53 (p-p53) or CHK2 (p-CHK2) (bar=100μm). Enlarged images of representative cells (marked with arrow) are also shown. The percentage of transduced melanocytes positive for p-p53 and p-CHK2 expression was quantitated from at least two independent transduction experiments from a total of at least 300 cells. The graph corresponds to the mean percentage of transduced cells treated with caffeine (+) or left untreated (-) ± s.d. (**D**) Human melanocytes were transduced with lentiviruses expressing N-RAS^Q61K^ or copGFP and cultured for 15 days in presence (+) or absence (-) of 4mM caffeine. The efficiency of transduction was controlled with the co-expression of copGFP and was consistently above 90%. Cell proliferation (Ki67), chromatin condensation (DAPI), and the appearance of increased SA-β-Gal activity were analyzed and quantitated 15 days after infection. Percentage of cells positive for the indicated marker is shown in histograms, which correspond to the mean ± s.d. of at least two independent transduction experiments from a total of at least 300 cells. Cells enlarged to show DAPI-stained chromatin foci are indicated with arrows (bar =10 μm). LM, light microscopy (bar=100μm).

N-RAS^Q61K^ induced melanocyte senescence was also associated with activation of the MAPK and AKT pathways, as shown by the increased phosphorylation of ERK (p-ERK), and AKT (p-AKT) at 5, 10 (data not shown) and 15 days post infection (Figure 1C). In addition, expression of oncogenic N-RAS led to p53 induction, increased expression of the p16^INK4a^ and p21^Waf1^ cyclin dependent kinase inhibitors and reduced accumulation of pRb phosphorylated at serine residues -807 and -811 (p-pRb) (Figure 1C). As previously reported, induced p14ARF was not detectable by Western blot analysis [34]. Oncogenic N-RAS also induced a robust DNA damage response in melanocytes that was associated with the accumulation of senescence-associated DNA damage foci, which contain phosphorylated histone H2AX (γ-H2AX) and are not equivalent to SAHF [15] (Figure 2A). Further, there was a marked increase in the phosphorylation of CHK2 on Thr-68 (p-CHK2) and increased p53 phosphorylation on Ser-15 (p-p53), two events associated with DNA damage (Figure 2B).

To examine the contribution of the DNA damage response to RAS-induced melanocyte senescence we suppressed ATM and ATR kinase activity with the addition of 4mM caffeine for 15 days. As expected, in the presence of N-RAS^Q61K^, the addition of caffeine markedly inhibited phosphorylation of the ATM targets CHK2 and p53 (Figures 2B, 2C). Nevertheless, suppression of the DNA damage response had no detectable impact on the N-RAS induced melanocyte senescence program. In particular, melanocytes accumulating N-RAS^Q61K^, regardless of exposure to caffeine, underwent potent cell cycle arrest (reduced Ki67 staining) that was associated with increased SA-β-Gal activity an the appearance of SAHF (Figure 2D). In addition, inhibition of the DNA damage checkpoint response did not impact on the N-RAS^Q61K^-mediated induction of total p53, p21^Waf1^, p16^INK4a^ and hypophosphorylated pRb (Figure 2B).

Considering that the p53 pathway remained active (increased p53 and p21^Waf1^ expression; see Figure 2B) in N-RAS^Q61K^-expressing melanocytes with a diminished DNA damage response, we examined whether oncogene-induced senescence of human melanocytes required the p53 protein. To silence p53 expression we utilised lentiviral shRNA vectors that specifically target p53 and to minimise confounding effects of shRNA off-target silencing two independent p53 silencing molecules were generated (Supplementary Figure 1). HEM1455 melanocytes were transduced with these shRNA molecules and three days post-infection the cells were re-transduced with lentiviral vectors expressing N-RAS^Q61K^ or copGFP. In all experiments we also applied a negative control shRNA molecule without homology to any human gene.

**Figure 3.**
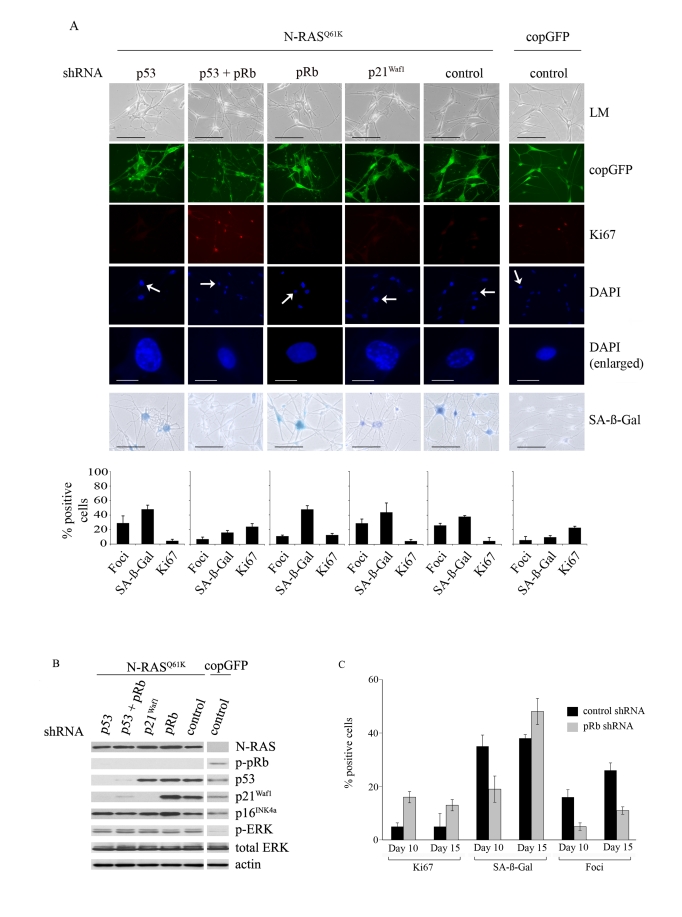
Relative contributions of the p53 and pRb tumour suppressor pathways in N-RAS ^Q61K^-induced melanocyte senescence. (**A**) Melanocytes were transduced with lentiviruses containing the indicated shRNA constructs. Three days post infection the cells were re-transduced with lentiviruses expressing N-RAS^Q61K^ or copGFP, as shown. Representative examples at 15days after infection are shown. Cell proliferation (Ki67), chromatin condensation (DAPI), and the appearance of increased SA-β-Gal activity were analyzed and quantitated. Percentage of cells positive for each indicated marker are shown in histograms, which correspond to the mean ± s.d. of at least two independent transduction experiments from a total of at least 300 cells. Cells enlarged to show DAPI-stained chromatin foci are indicated with arrows (bar =10 μm). LM, light microscopy (bar=100μm). (**B**) Expression of the indicated proteins was determined by western blot analysis at 15 days after infection of human epidermal melanocytes with the indicated shRNA constructs and either lentivirus expressing N-RAS^Q61K^ or the copGFP control. (**C**) The impact of pRb-silencing on the N-RAS^Q61K^ induced senescence was determined by quantitating key senescence markers (Ki67 expression, SAHF formation, SA-β-Gal activity) at 10 and 15 days post N-RAS transduction. Percentage of cells positive for each indicated marker is shown in histograms, which correspond to the mean ± s.d. of at least two independent transduction experiments from a total of at least 300 cells.

The inhibition of p53 expression did not alter the cell cycle arrest induced by oncogenic N-RAS^Q61K^ (15 days after infection only 5% of N-RAS^Q61K^ melanocytes showed positive Ki67 staining regardless of p53 expression and this can be compared to 23% Ki67 positive p53-null melanocytes infected with the copGFP control; Figure 3A). Similarly, cellular senescence was initiated and maintained in the presence or absence of p53 expression; increased SA-β-Gal activity appeared in 48% of p53-null cells compared to 38% in the p53-positive control cells, 15 days post transduction (Figure 3A) and the two different p53-specific shRNAs exerted similar effects (data not shown). In fact no markers of senescence, including cell morphology, SA-β-Gal activity, appearance of SAHF or Ki67 incorporation discriminated between p53-intact and p53-null senescent melanocytes. It is important to note, however, that p21^Waf1^ expression was not induced by oncogenic N-RAS in p53-deficient melanocytes (Figure 3B).

In p53-null N-RAS melanocytes the induction of p16^INK4a^ and hypophosphorylation of pRb was maintained (Figure 3B), and it seemed likely that the activation of pRb was dominant and sufficient to establish melanocyte senescence. Certainly silencing expression of both p53 and pRb bypassed N-RAS induced cell cycle arrest and senescence in this cell type (15 days after infection only 5% of N-RAS^Q61K^ melanocytes showed positive Ki67 staining, compared to 24% of N-RAS^Q61K^ melanocytes lacking both p53 and pRb and 23% of melanocytes expressing only control shRNA/copGFP; Figure 3A). To examine the individual role of pRb, HEM1455 melanocytes were transduced with a pRb-specific shRNA molecule and three days post-infection the cells were re-transduced with lentiviral vectors expressing N-RAS^Q61K^ or copGFP. pRb-null melanocytes responded to oncogenic N-RAS with delayed onset of cell cycle arrest and senescence. In particular, 10 days post infection with oncogenic N-RAS, 16% of pRb-null melanocytes remained positive for the proliferation marker Ki67 compared to only 5% of the pRb-positive melanocytes. Similarly, SA-β-Gal activity was detected in only 19% of pRb-deficient N-RAS^Q61K^ melanocytes compared to 35% in the pRb-positive N-RAS^Q61K^ cells. Further, the percentage of N-RAS^Q61K^ expressing cells with SAHF was clearly reduced, and remained so in the absence of pRb (Figure 3C).

These data suggest that the activation of pRb is the dominant effector of oncogene-induced melanocyte senescence, and thus upstream regulators of pRb function may represent critical melanoma tumour suppressors. For instance, loss of the melanoma predisposition gene p16^INK4a^, detectably weakened the pRb-pathway and the senescence program in melanocytes by inhibiting the pRb-dependent development of SAHF [34]. Considering that the CDK inhibitors p16^INK4a^ and p21^Waf1^ were both potently induced in melanocytes in response to N-RAS^Q61K^ expression (see Figure 1C), we wanted to establish whether the function of p16^INK4a^ in the formation of SAHF was specific to this CDK inhibitor or whether another senescence-associated CDK inhibitor p21^Waf1^ was equivalent in activity. The role of p21^Waf1^ was examined utilising two, highly effective p21^Waf1^-specific lentiviral shRNA vectors (Supplementary Figure 1). HEM1455 melanocytes were transduced with these shRNA molecules and three days post-infection the cells were re-transduced with lentiviral vectors expressing N-RAS^Q61K^ or copGFP. In all experiments we also applied a negative control shRNA molecule without homology to any human gene.

Depletion of p21^Waf1^ did not detectably alter N-RAS induced cell cycle arrest or senescence in human melanocytes. The p21^Waf1^-deficient melanocytes responded to oncogenic N-RAS by accumulating hypo-phosphorylated pRb, p16^INK4a^ and p53 (Figure 3B), they enlarged, acquired increased SA-β-Gal activity and were negative for the proliferation marker Ki67 (Figure 3A). Unlike pRb-null melanocytes, there was no detectable delay in N-RAS induced arrest and senescence in p21^Waf1^-deficient melanocytes. Importantly, in the absence of the p21^Waf1^ CDK inhibitor, the formation of SAHF was not altered 10 and 15-post transduction (29% foci in p21^Waf1^-null, vs 11% foci in pRb-null vs 26% foci in shRNA control cells, 15 days post infection with N-RAS^Q61K^; Figure 3A). The second p21^Waf1^-specific shRNA exerted similar effects (data not shown).

**Figure 4.**
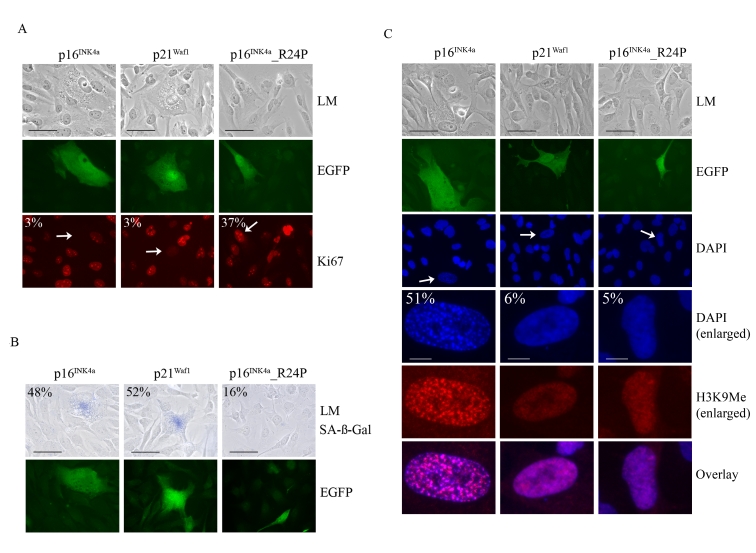
Impact of p16 ^INK4a^ or p21^Waf1^ expression on the cellular senescence program. WMM1175 melanoma cells were cotransfected with plasmids encoding p16^INK4a^, p21^Waf1 ^or the melanoma-associated p16^INK4a^_R24P along with *pCMV-EGFPN1*, which was used as a marker of transfection. Five days post transfection cells were fixed, permeabilized and analyzed. (**A**) Cell proliferation was monitored by Ki67 immunostaining and the percentage of transfected WMM1175 cells with positive Ki67 staining is indicated and was determined from at least two separate transfection experiments and from a total of at least 300 cells. All standard deviations were less than ±5% (bar=100μm). (**B**) Transfected WMM1175 cells were analyzed for SA-β-Gal activity, and the percentage of positive SA-β-Gal transfected cells is indicated, and was determined as detailed above (bar=100μm). (**C**) The appearance of SAHF was analyzed by immunostaining with antibodies to H3K9Me and co-staining DNA with DAPI. The percentage of transfected cells with detectable foci is indicated, and was determined as detailed above (bar=100μm).

To further investigate whether p16^INK4a^ was unique in promoting SAHF formation we developed a transient melanoma model to rapidly assess the functions of the p21^Waf1^ and p16^INK4a^. The functionally impaired p16^INK4a^_R24P mutant that is unable to bind and inhibit CDK4 but retains CDK6 inhibitory activity was used as a control [34,39]. The WMM1175 melanoma cells were transiently transfected with plasmids encoding each of these CDK inhibitors along with a plasmid encoding the enhanced green fluorescent protein (EGFP), which was used as a marker of transfection.  The cell cycle proliferation, SAHF formation and SA-β-Gal activity of transfected WMM1175 cells was then assessed over 5-days. This was enough time to observe the induction of senescence and protein expression from the transiently transfected plasmids was still detectable. As expected, ectopic expression of wild-type p16^INK4a^and p21^Waf1^, but not p16^INK4a^_R24P promoted rapid cell cycle arrest (Figure 4A). Similarly, p16^INK4a^ and p21^Waf1^ but not the R24P variant induced cell enlargement, and increased SA-β-Gal activity by five days post transfection (Figure 4B). The only detectable difference between the two wild type CDK inhibitors was the induction of SAHF; only p16^INK4a^ accumulation led to the appearance of these distinctive foci, which were enriched for H3K9Me (Figure 4C).

## Discussion

The molecular mechanisms that trigger oncogene-induced senescence have been studied extensively, and yet the relative contribution of the p16^INK4a^/pRb and the p53/p21^Waf1^ pathways in initiating and maintaining the senescence program remains poorly understood. In this study, we show that N-RAS^Q61K^induces senescence in human melanocytes that was associated with markers of DNA damage response, and involved the activation of both the p53 and pRb pathways. Surprisingly neither the pharmacological inhibition of the DNA damage response pathway with caffeine nor silencing of p53 expression had a detectable impact on the N-RAS^Q61K^induced senescence of human melanocytes. In fact, no markers of senescence, including cell morphology, SA-β-Gal activity, appearance of SAHF or Ki67 incorporation discriminated between p53-intact and p53-null senescent melanocytes. Interestingly, caffeine diminished the phosphorylation of p53 on Ser-15, but did not reduce the overall levels of p53, or its activity (as measured by p21^Waf1 ^induction; Figure 2B) in melanocytes. Several other reports have also shown that inhibition of p53 phosphorylation at Ser-15 did not correlate with diminished p53 activity and this is indicative of p53 stabilization via multiple mechanisms [40,41]. It is tempting to suggest that the melanoma tumour suppressor p14ARF is the critical activator of p53 in melanocytes. p14ARF stabilizes p53 by binding and inhibiting the p53 specific ubiquitin ligase, mdm2 [42], rather than inducing p53 phosphorylation. We have previously shown however that p14ARF is only weakly induced by oncogenic N-RAS in human melanocytes, and is not required for p53 activation in response to N-RAS [34]. In fact, the ARF tumour suppressor appears to contribute to oncogene-induced senescence only in mouse cells (Table 1).

It is reasonable to assume that in the absence of p53 the activated p16^INK4a^/pRb pathway was sufficient to initiate and maintain senescence, and this appears to be the case in melanocytes. Not only did oncogenic N-RAS potently induce p16^INK4a^ in melanocytes, pRb existed in its active hypophosphorylated form, and silencing of pRb significantly delayed the onset of senescence. Ultimately, the senescence program was activated in pRb-null melanocytes and this required the p53 pathway, as the simultaneous loss of p53 and pRb completely overcame N-RAS induce senescence in melanocytes. This is the first demonstration showing that melanocytes senesce in response to oncogenic signaling by engaging both the p53 and pRb pathways.

It has been suggested that p53, p21^Waf1^ and pRb act in a linear pathway, with p53-induced p21^Waf1^ activating pRb to regulate cell entry into replicative senescence [43]. This model does not adequately account for the fact that pRb-null melanocytes ultimately senescence in response to oncogenic N-RAS. It is possible that the pRb homologues, p107 and p130 participate in oncogene-induced senescence as they can functionally compensate for pRb loss and, like pRb, are activated by p21^Waf1^and p16^INK4a^[44]. Certainly, pRb-deficient MEFs senesce in culture, whereas MEFs with targeted deletion of all three pRb family members (pRb, p107 and p130) do not [45]. Furthermore, p53 was capable of inducing senescence in pRb-null prostate cancer cells, but not in p107 and pRb depleted cells [46]. Although such compensation clearly exists, the fact that pRb mutations are common in human cancer, whereas p107 and p130 mutations occur rarely [47], suggests that functional compensation for pRb loss must be context dependent. In the case of melanocytes, pRb (not p107 and p130) is required for normal mouse melanocyte proliferation although arrest in response to growth factor deprivation was associated with the formation of pRb- and p130-transcription repressor complexes in human melanocytes (reviewed in [48]). We are currently exploring whether the response of human melanocytes to oncogenic signalling involves the pRb homologues, p107 and p130 and whether the contribution of p53 to melanocyte senescence is strictly dependent on the pRb family of proteins.

Our data clearly demonstrate that oncogenic N-RAS acts primarily through the pRb pathway in melanocytes. Activation of this pathway involves both p21^Waf1^ and p16^INK4a^, and these were the only CDK inhibitors potently induced by oncogenic N-RAS in melanocytes (data not shown). We confirm that both p16^INK4a^ and p21^Waf1^ can induce senescence, but their activities are clearly distinct. p16^INK4a^ expression promoted the formation of DAPI-stained heterochromatin foci that were enriched for the H3K9Me marker of SAHF. In contrast, ectopic p21^Waf1^ expression had no detectable impact on chromatin structure even though cells were clearly arrested. Similarly, loss of p16^INK4a^ reduced the formation of SAHF in melanocytes [34], whereas loss of p21^Waf1^, either via direct silencing or by silencing p53, had no detectable effect on SAHF formation. Although both p16^INK4a^ and p21^Waf1^ can activate pRb their actions are not equivalent. p16^INK4a^ is a potent inhibitor of the cyclin D-dependent kinases, CDK4 and CDK6, whereas p21^Waf1^ is sequestered by and acts as a positive regulator of these kinases. This pool of tethered p21^Waf1^ is released as p16^INK4a^ accumulates and p21^Waf1^ redistributes to bind and inhibit cyclin E-CDK2 complexes and induce G1 arrest [49]. The ability of p16^INK4a^ to inhibit the cyclin D-dependent kinases also enables it to block the assembly of DNA replication complexes onto chromatin and thus inhibit DNA replication, a function not shared by p21^Waf1 ^[50]. Thus, in melanocytes with oncogenic signalling only p16^INK4a ^can fully engage the pRb pathway to alter chromatin structure and silence the genes that are required for proliferation. Melanocytes undergoing replicative senescence also rely on the p16^INK4a^/pRb axis, as p53 and p21^Waf1^ levels remain low in these arrested melanocytes [27]. We suggest that inhibition of cyclin D-dependent kinases and induction by senescence-causing stimuli necessitate p16^INK4a^ inactivation in human cancers and distinguish this CDK inhibitor as a tumour suppressor.

## Materials and methods

Cell culture and transfections . Human WMM1175 melanoma cells (ARF-null, p53-null, pRb+/+; [51]) and U20S osteosarcoma cells were grown in Dulbecco'smodified Eagle's medium (DMEM, Gibco BRL, Carlsbad, CA,USA) supplemented with 10% foetal bovine serum andglutamine. Human epidermal melanocytes (HEMs) were obtained from Cell Applications (Cell Applications, San Diego, CA, USA) and grown in HAM's F10 media (Sigma, St. Louis. MO, USA), supplemented with ITS premix (Becton Dickinson, Franklin Lakes, NJ, USA), TPA, IBMX, cholera toxin, 20% fetal bovine serum and glutamine (modified from [52]). All cells were cultured in a 37°C incubator with 5% CO_2_. Caffeine (Sigma) was used at 4mM for 15 days.

For *p16^INK4a^, p21^Waf1^, p16^INK4a^_R24P* transfections, WMM1175 cells (1 × 10^5^)were seeded on coverslips in six-well plates and transfected with2μg plasmid encoding p16^INK4a^, p21^Waf1^, or p16^INK4a^_R24P and 100ng *pEGFPN1 *(Clontech, Mountain View, CA, USA), as a transfection marker, usingLipofectamine 2000 (Invitrogen, Carlsbad, CA, USA).

Lentivirus transductions . Lentiviruses were produced in HEK293T cells using the *pSIH1-H1-copGFP* (Copepod green fluorescent protein) shRNA expression vector or the *pCDH-CMV-MCS-EF1-copGFP* lentiviral vector (Systems Biosciences, Mountain View, CA, USA) encased in viral capsid encoded by three packaging plasmids as described previously [53]. Viruses were concentrated as described previously [54]. Viral titres were determined using 1 x 10^5^ U2OS cells/well in six-well plates, transduced with serial dilutions of the concentrated viral stocks in the presence of Polybrene (8 μg/ml; Sigma). Cells were harvested 48 h post-transduction, analysed by flow cytometry for GFP expression and viral titre calculated. Cells were infecting using an MOI of 5-10 to provide infection efficiency above 90%.

Constructs . The *N-RAS* lentiviral construct and p16^INK4a^ plasmids have been described elsewhere [33,55]. The p21^Waf1^ cDNA was kindly provided by Dr B. Vogelstein and subcloned into the *pFLAG-CMV5b* mammalian expression vector (Sigma). The p53-directed shRNA sequences correspond to nucleotides 956-974 and 1026-1044 [56,57] (Genbank accession number NM_000546). The p21^Waf1^-directed shRNA sequences correspond to nucleotides 560-578 and 569-587 (Genebank accession number NM_078467) [58]. The shRNA sequence targeting pRb corresponded to nucleotides 662-680  (Genebank accession number NM_000321.1) [59]. The non-silencing negative control shRNA did not show complete homology to any known human transcript and had the following sequence: 5'-TTAGAGGCGAGCAAGACTA-3'.

Western blotting . Total cellular proteins were extracted at 4°C using RIPA lysis buffer containing protease inhibitors (Roche, Basel, Switzerland). Proteins (30-50μg) were resolved on 12% SDS-polyacrylamide gels and transferred to Immobilon-P membranes (Millipore, Bedford, MA, USA). Western blots were probed with antibodies against p16^INK4a^ (N20, Santa Cruz, CA, USA), p21^Waf1^ (C-19, Santa Cruz), β-actin (AC-74, Sigma-Aldrich), p53 (DO-1, Santa Cruz), p-p53 (#9284, Cell Signalling, Danvers, MA, USA), p-ERK (E4, Santa Cruz), ERK (137F5, Cell Signalling), p-AKT (L32A4, Cell Signalling), AKT (11E7, Cell Signalling), c-MYC (A14, Santa Cruz), H3K9Me (Millipore) and phosphorylated p-pRb (#9308, Cell Signalling).

Indirect immunofluorescence . Cultured cells (3-4 x 10^4^) seeded on coverslips in 12-well plates were washed in PBS and fixed in2% formaldehyde, 0.2% glutaraldehyde, 7.4 mM Na_2_HPO_4_, 1.47 mM KH_2_PO_4_, 137 mM NaCl, and 2.68 mM KCl. Cells were then rinsed three times with PBS and SA-β-Gal activity was detected as previously described [60]. Cells fixed in 3.7% formaldehyde were immunostained for 50 min with primary antibody followed by a 50 min exposure to Alexa Fluor 594-conjugated secondary IgG  (Molecular Probes, Carlsbad, CA, USA).

## Supplementary figure

Supplementary Figure 1 Lentiviruses containing the indicated shRNA constructs cloned into the *pSIH-H1-copGFP* vector (System Biosciences) were used to infect the U20S osteosarcoma cells. Approximately three-four days post infection, p21^Waf1^, p53 and pRb protein expression was analysed by western blot as indicated.
