# Supplementary figures and images for: The relative contributions of the p53 and pRb pathways in oncogene-induced melanocyte senescence
Source: Aging (Albany NY). 2009 May 16;1(6):542–56. doi: 10.18632/aging.100051 (PMC2806033; doi:10.18632/aging.100051)

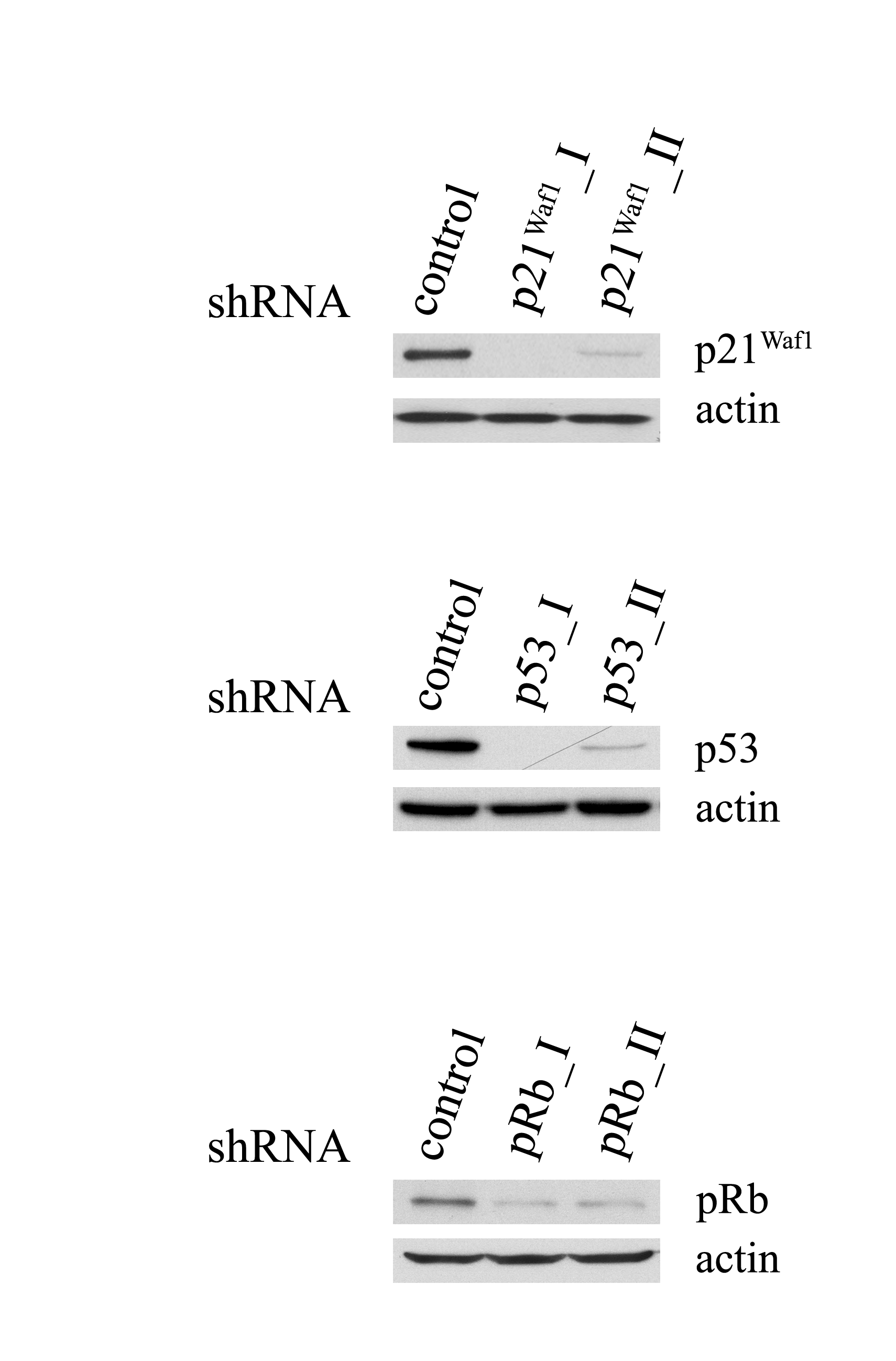

Supplement: Supplementary Figure 1 — Lentiviruses containing the indicated shRNA constructs cloned into the pSIH-H1-copGFP vector (System Biosciences) were used to infect the U20S osteosarcoma cells. Approximately three-four days post infection, p21Waf1, p53 and pRb protein expression was analysed by western blot as indicated. [file aging-01-542-s001.tif]
